# IS THERE A RELATION BETWEEN HELYBACTER PYLORI AND INTESTINAL METAPLASIA IN SHORT COLUMN EPITELIZATION UP TO 10 MM IN THE DISTAL ESOPHAGUS?

**DOI:** 10.1590/0102-672020190001e1480

**Published:** 2019-12-20

**Authors:** Matheus DEGIOVANI, Carmem Australia Paredes Marcondes RIBAS, Nicolau Gregori CZECZKO, Artur Adolfo PARADA, Juliana de Andrade FRONCHETTI, Osvaldo MALAFAIA

**Affiliations:** 1 Postgraduate Program in Principles of Surgery, Mackenzie Evangelical School of Medicine - Paraná, Curitiba, PR, Brazil; 2 Digestive Endoscopy Service, 9 de Julho Hospital, São Paulo, SP, Brazil

**Keywords:** Barrett’s esophagus, Helicobacter pylori, Intestinal metaplasia, Esôfago de Barrett, Helicobacter pylori, Metaplasia intestinal

## Abstract

**Background:**

The presence of intestinal metaplasia in the distal esophagus (Barrett’s esophagus) is an important precursor of adenocarcinoma. Knowledge of the risk factors and the process by which the Barrett develops is very important and *Helicobacter pylori* (HP) can contribute to this development.

**Aim:**

To analyze the impact of HP in the gastric mucosa with intestinal metaplasia in the distal esophagus in areas of columnar epithelialization smaller than 10 mm in length and epidemiological data on prevalence

**Method:**

A retrospective study in which were included 373 consecutive patients diagnosed with columnar epithelium in the distal esophagus was done. In all, HP was investigated by urease and histology, exclusion and inclusion factors were applied and patients were divided into two groups: the first grouping the ones without histological diagnosis of Barrett’s esophagus (235-63%) and the second with it (138-37%).

**Results:**

There was no significant difference between HP and non-HP patients in relation to the probability of having intestinal metaplasia (p=0.587). When related to the general group, there was an inverse association between the bacterium and the columnar epithelia in the distal esophagus. Age (p=0.031), gender (p=0.013) and HP (p=0.613) when related together to intestinal metaplasia showed no significant relation. In isolation, when related to age and gender, regardless of HP, results confirmed that patients in more advanced age and women present a higher incidence of intestinal metaplasia.

**Conclusion:**

There is an inverse relation between HP and the areas of columnar epithelization in the distal esophagus, regardless of the presence or absence of intestinal metaplasia. Age and gender, regardless of HP, showed higher prevalence in women and in older the number of cases with intestinal metaplasia in the distal esophagus.

## RESUMO

**Racional:**

A presença de metaplasia intestinal no esôfago distal (esôfago de Barrett) é importante doença precursora do adenocarcinoma. O conhecimento sobre os fatores de risco e o processo pelo qual ela se desenvolve é importante e o *Helicobacter pylori* (HP) pode contribuir para esse desenvolvimento.

**Objetivo:**

Analisar o impacto do HP na mucosa gástrica sobre a metaplasia intestinal no esôfago distal em áreas de epitelização colunar menores que 10 mm de extensão e dados epidemiológicos de prevalência.

**Método:**

Estudo retrospectivo com inclusão de 373 pacientes consecutivos, com diagnóstico de epitélio colunar no esôfago distal. Em todos foi pesquisado o HP pela urease e histologia, aplicados os fatores de exclusão e inclusão e divididos em dois grupos: o primeiro agregando os pacientes sem diagnóstico histológico de esôfago de Barrett (235-63%) e o segundo com ele (138-37%).

**Resultados:**

Não houve diferença significativa entre os portadores ou não do HP em relação à probabilidade de ter metaplasia intestinal (p=0,587). Quando relacionado ao grupo geral, houve associação inversa entre a bactéria e a epitelização colunar em esôfago distal. A idade (p=0,031), gênero (p=0,013) e HP (p=0,613) quando relacionados juntos à metaplasia intestinal não mostraram relação significativa. Isoladamente, quando relacionados idade e gênero, independente do HP*,* surgiram resultados confirmando que pacientes de idade mais avançada e mulheres apresentam maior incidência de metaplasia intestinal.

**Conclusão:**

Existe relação inversa entre HP e as áreas de epitelização colunar em esôfago distal, independente da presença ou não de metaplasia intestinal. Já em relação à idade e gênero, independente do HP*,* notou-se que em mulheres e com maior a idade há aumento no número de casos com metaplasia intestinal no esôfago distal.

## INTRODUCTION

Strong evidence has indicated that *Helicobacter pylori* (HP) infection plays an important role in the pathogenesis of digestive tract diseases^26^ and among them is esophageal adenocarcinoma^1^ ^,^ ^3^ ^,^ ^27^. Its incidence has increased over three decades in developed countries, while the five-year survival rate remains low^10^ ^,^ ^36^ There are precursor factors for its development^19^ ^,^ ^21^, being Barrett’s esophagus (EB) the principle, whose incidence is relatively high compared to other precursors of malignancy in western populations.

In the American school, EB is considered when there is gastric mucosa in the esophagus (proximal displacement of the squamocolumnar junction in relation to the esophagogastric junction), whose biopsies make the diagnosis of intestinal metaplasia, i.e., the presence of goblet cells in the anatomopathological study^35^

The British and Japanese societies of gastroenterology define it by the presence of columnar epithelium in areas of esophageal mucosa, without the need for goblet cells for its diagnosis. They state that EB without intestinal metaplasia is “biologically intestinalized” and has molecular changes similar to those of EB with goblet cells^15^ screening and diagnosis, surveillance, pathological grading for dysplasia, management of dysplasia, and early cancer including training requirements. The rigour and quality of the studies was evaluated using the SIGN checklist system. Recommendations on each topic were scored by each author using a five-tier system (A+, strong agreement, to D+, strongly disagree.

According to the Brazilian Society of Digestive Endoscopy (SOBED), the diagnosis of EB is made through endoscopy with biopsy of the area appearing to be gastric epithelium in the distal esophagus (columnar epithelialization). For its confirmation, the presence of intestinal metaplasia in the esophageal biopsy is required. It should not be done in the presence of active esophagitis (erosions), as it can hide EB under erosions or inflammation by mimicking dysplasia and altering the pathological diagnosis.

 The relationship between HP and diseases related to esophageal acid reflux is uncertain^2^ ^,^ ^23^. It is currently advocated that the presence of this bacterium in the gastric mucosa may trigger a protective effect for patients with gastroesophageal reflux disease (GERD), both for esophagitis and for the development of EB and esophageal adenocarcinoma. This happens because HP gastric colonization decreases stomach acid secretion, leading to decreased gastroesophageal acid reflux aggression^18,20^ 906 city residents during a health-screening programme between 1974 and 1992. Forty-four cases of oesophageal cancer and 149 matched controls were selected. The mean interval between screening and cancer diagnosis was 11.9 years. H. pylori seropositivity was determined by an enzyme-linked immunosorbant assay measuring IgG. Occupation was included in the statistical analysis as an indicator of socio-economic status.\\n\\nRESULTS: Helicobacter pylori seropositivity was present in 10 of the cases (22.7%

The objective of this study was to correlate intestinal metaplasia to HP infection in areas of distal esophageal columnar epithelialization less than 10 mm in length, and it´s connection to age and gender.

## METHOD

The research project was approved by the Research Ethics Committee of the Mackenzie Evangelical College of Paraná, Curitiba, PR, Brazil and registered with Plataforma Brasil. It was performed at the Gastrointestinal Endoscopy Service of Hospital Nove de Julho, São Paulo, SP, Brazil with consecutive patients who underwent upper digestive endoscopy. 

The initial sample consisted of 14,894 patients who underwent upper digestive endoscopy. Of this total, 373 were selected and divided into two groups. The first included those with endoscopic diagnosis of columnar epithelialization without intestinal metaplasia (n=235), and the second with epithelialization (n=138). All were stratified by gender and age.

In both groups gastric biopsies were performed to search for PH using two methods: histopathological and urease. For histopathological study they were collected at the sites recommended by the Houston Consensus: two fragments of the antrum close to the pylorus, one in incisura angularis and two in gastric body (small and large gastric curvature)^9^. For urease, Uretest (Renylab®, MG) and biopsies were performed with at least two antrum fragments, one from incisura angularis and two from the body^5^. Tweezers used for material collection were disposable (Boston®) with a 6 mm opening.

**FIGURE 1:**
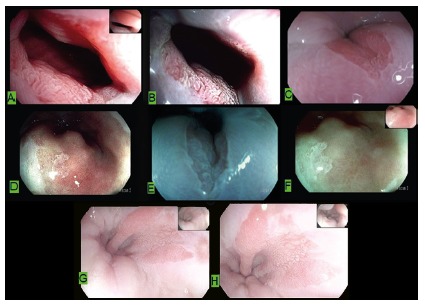
Areas of distal esophageal columnar epithelialization delimited with acetic acid and digital chromoscopy: A) magnified with acetic acid; B) magnified with digital chromoscopy; C) with acetic acid; D, E and F) with different digital chromoscopies; G and H) with acetic acid.

The patients underwent videoendoscopy with high-definition devices (Pentax i scan) using up to 2x optical zoom, digital chromoscopy and acetic acid to better identify areas of columnar epithelium and direct biopsies (Figure 1). The images obtained were recorded and saved in a database (Perseus and Endox® system, SP). At least 10 images were obtained per patient in all cases. 

**FIGURE 2:**
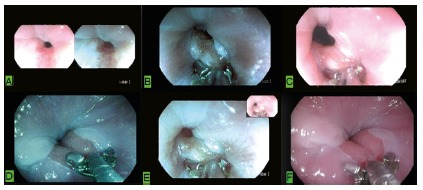
Targeted biopsies with acetic acid and digital chromoscopy in areas of columnar epithelium smaller than 10 mm: A) areas less than 10 mm to be biopsied (acetic acid and digital chromoscopy); B, C, D and F) boundaries of areas to be biopsied with digital chromoscopy.

All enrolled patients underwent endoscopy for the first time. The only inclusion criterion was the diagnosis of columnar epithelium in the distal esophagus less than 10 mm in length. Exclusion criteria were grade B, C and D esophagitis in the Los Angeles (1994) classification, inadequate biopsies, insufficient sedation during the examination, previous treatment for PH, and patients for whom biopsy was not recommended (Figure 2).

### Statistical analysis

The variables HP, gender and intestinal metaplasia were presented in frequencies and percentages. In comparing the groups defined by the presence or absence of metaplasia in relation to age, Student’s t test for independent samples was used. To evaluate the association between gender and presence of PH with metaplasia, Fisher’s exact test was used. Age results were described by means, medians, minimum values, maximum values and standard deviations. For multivariate analysis of factors associated with metaplasia, the logistic regression model was adjusted followed by the Wald test. Values of p<0.05 were statistically significant. Data were analyzed using the IBM SPSS Statistics v.20 program.

## RESULTS

Of the 373 cases, 138 (37%, 95% CI: 32.1% to 41.9%) were diagnosed with intestinal metaplasia and 235 (63%) without. The HP was negative in 337 cases (90.3%) and positive in 36 (9.7%, 95% CI: 6.7% to 12.6%).

### Evaluation of the association between PH and columnar esophagus

The test result indicates the rejection of the null hypothesis (p<0.001) in this comparison. The percentage of negative cases was significantly higher than 50% (95% CI: 87.4% to 93.3%). There was an inverse relationship between PH and columnar esophagus.

### Relationship between PH and intestinal metaplasia

The results indicated no significant difference between patients with and without HP regarding the probability of having intestinal metaplasia. Among patients with HP+ this percentage was 41.67%. This difference is not statistically significant (Table 1).

**TABLE 1:** Data relating HP to intestinal metaplasia

Intestinal metaplasia	H.pylori	
	Negative	Positive
Positive (EB)	123	15
	36.50%*	41.67%*
Negative	214	21
	63.50%	58.33%
Total	337	36

*=p 0.587

### Age and gender assessment and diagnosis of intestinal metaplasia

**FIGURE 3:**
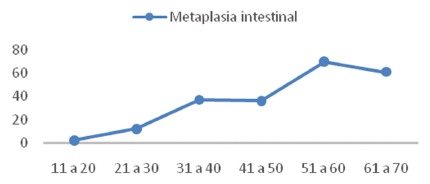
Age vs. intestinal metaplasia

Regarding the mean age, the results indicated a significant difference between patients with and without metaplasia. Those with on average were three to four years older than those without this diagnosis (Table 2, Figure 3).

**TABLE 2:** Age related data

Metaplasia	Age						p*
	n	Average	Median	Minimun	Máximun	Standart Derivation	
No	235	50.3	52.0	16.0	89.0	14.4	
Yes	138	53.7	56.0	16.0	88.0	13.6	0.025

*=Student’s t-test for independent samples, p<0.05

Regarding gender, the results indicated a significant difference in the probability of diagnosing intestinal metaplasia. Women were more likely to be diagnosed than men: men n=44 (29.14%) and women 42.53% (Table 3).

**TABLE 3:** Gender-related data

Intestinal metaplasia	Gender	
	Male	Female
Positive	44	94
	29.14%*	42.53%*
Negative	107	127
	70.86%	57.47%
Total	151	221

*p=0,009

### Multivariate analysis

To assess the effect of the presence of HP on the likelihood of having metaplasia in gender and age, a logistic regression model was adjusted. The results indicated that, adjusting for these variables, no significant associations were found (Table 4).

**TABLE 4:** Statistical data between associations of risk factors for intestinal metaplasia

Variable	p	OR (IC95%)
Age	0.031	1.017 (1.001 a 1.033)
Gender	0.013	1.76 (1.13 - 2.76)
H. pylori	0.613	1.20 (0.59 - 2.46)

Regarding age, regardless of gender and the presence or absence of HP, a significant association was found with intestinal metaplasia. It is estimated that each year over age there is an increase of 1.7% in OR onset. For gender, regardless of age and the presence or absence of HP, a significant association with intestinal metaplasia was found. The OR for intestinal metaplasia in women was 20% higher than in men.

## DISCUSSION

The correlation between HP and Barrett’s esophagus is debatable in the literature. There are aspects regarding the effect of gastric colonization by it on gastroesophageal reflux disease, Barrett’s esophageal development and its correlation with esophageal adenocarcinoma. To this end, there are studies that report HP as a risk factor for Barrett’s esophagus, while others propose the unrelation between them^29^ ^,^ ^32^ and also those that infer its protective effect^6^ ^,^ ^11^.

In this study, when HP was related to columnar epithelializations in the distal esophagus, a significant inverse relationship was obtained. At the same time, when HP is related to the presence or absence of intestinal metaplasia in these areas, there was no significance. Studies showing an inverse relationship between HP and EB necessarily do so with the areas of distal esophageal columnar epithelialization, developed from gastroesophageal reflux, on which biopsies are performed for the diagnosis of EB. Thus, in this series, it can be stated that there is a significant inverse relationship between HP and diseases related to gastric acid reflux, i.e., columnar epithelializations in the distal esophagus.

Thus, another important point to be considered is that if we think about the definitions of EB by the Brazilian Society of Digestive Endoscopy and the American school, we would have a mismatch between the relationship between HP and EB. However, according to the Japanese and British school, who consider Barrett’s epithelium without intestinal metaplasia to be “biologically intestinalized,” there is an inverse or “protective” relationship between HP and EB.

In accordance with this hypothesis of protection of HP from GERD and columnar epithelializations in the distal esophagus, Xue et al.^39^ they were divided into H. pylori positive and negative groups. H. pylori positive patients were randomly given H. pylori eradication treatment for 10 days, then esomeprazole 20 mg bid for 46 days. The other patients received esomeprazole 20 mg bid therapy for 8 weeks. After treatment, three patient groups were obtained: H. pylori positive eradicated, H. pylori positive uneradicated, and H. pylori negative. Before and after therapy, reflux symptoms were scored and compared. Healing rates were compared among groups. The χ^2^ test and t-test were used, respectively, for enumeration and measurement data.\\n\\nRESULTS: There were 176 H. pylori positive (with 92 eradication cases studied 846 patients with reflux esophagitis who underwent treatment with pantoprazole (40 mg). After four weeks of treatment the reflux esophagitis healing rate was 86.6% in patients with HP positive. In patients with HP negative, healing was 76.1%. After eight weeks the healing rates in the positive and negative groups were 96.4% and 91.8% respectively. Thus, healing in the HP positive group was higher when compared to the HP negative group, suggesting that, based on the same treatment, better results were obtained in patients diagnosed with HP.

According to numerous publications, it is clinically relevant that in patients with GERD and its complications, the prevalence of HP is lower. As an example, in a systematic review, the average prevalence of infection in patients with GERD was 38.2%, compared to 49.5% of patients with GERD without HP^30^. Others show a protective effect exerted by HP on GERD, triggering less severe forms of the disease in infected patients^16^ ^,^ ^37^.

In accordance with this protective theory of HP against esophageal diseases, Rubenstein et al.^33^ demonstrated that the higher the level or severity of esophagitis - according to pre-established endoscopic grade I, II and III grading - the prevalence of HP is lower (51.72%, 28.57%, 20.68% respectively), regardless of the presence or absence of EB.

In an attempt to justify or not the relationship between HP and esophageal diseases, several meta-analyses were performed. However, in all, there are conclusive reports about the difficulties of their accomplishments in face of regional adversities, heterogeneities of existing studies and information bias in data collection.

Thus, in Wang et al.^38^ meta-analysis for example, there was significant heterogeneity, which was explained by the type of control group selected in some studies and studies conducted in Asia, where the incidence HP is superior to Western countries, concluding that further study is needed.

In another meta-analysis, Fischback et al.^19^ in 49 studies suggest that the presence of HP in the gastric mucosa is associated with reduced risk of EB. However, despite the difficulties already reported for performing meta-analyses on this subject, four of these 49 studies, with no bias, showed a reduced risk of EB in HP infected patients, including Corley et al.^7^ in California and Fischback et al.^14^ in Ireland. These same authors after two years, in a case-control study associating HP and EB, concluded that the presence of the bacteria was inversely associated with the presence of EB. The chance of HP infection in the gastric mucosa in EB patients has been found to be 50% lower when compared to patients without EB^12^.

Following the same principle, Rokkas et al.^31^ in meta-analysis showed as statistically significant the inverse association between HP and esophageal adenocarcinoma, developed on EB. In contrast, in patients with squamous cell carcinoma there was no significant relationship with the prevalence of HP. 

Rubenstein et al.^34^ concluded that in patients with erosive esophagitis and EB the prevalence of HP is lower. In the same study, there was no relevance among symptomatic patients for GERD and HP, contributing to the hypothesis that their infection does not protect against EB itself, but decreases GERD.

Contrary to this hypothesis, Yaghoobi et al.^40^ English, multiple-source literature search was performed from 1983 to February 2007. Only randomized controlled trial (RCT in meta-analysis revealed that erradication of HP usually does not promote GERD. Based on this assumption, Hackelsberger et al.^17^whether in the cardia or the distal oesophagus, has been uniformly defined as specialised columnar epithelium, suggesting a relation with Barrett’s oesophagus. It is, however, not clear whether the risk factors associated with intestinal metaplasia are identical at both sites.\\n\\nAIMS: To investigate biopsy specimens obtained below the squamocolumnar junction (SCJ contradicted the protective hypothesis of HP to EB, when they reported that the pathogenesis of esophageal intestinal metaplasia is not uniform and may be related to several other factors.

Therefore, there are other possibilities of relating HP and EB. As an example, a direct systemic effect would be the action of a specific type of HP DNA causing decreased regulation of Interferon type 1 and Interleukin 12 responses to inflammatory stimuli, facilitating the development of esophagitis and its complications. Another possibility of improvement of esophageal diseases in which HP could act indirectly may be related to the effect mediated by the ghrelin peptide. When there is a basal decrease in secretion, there is a concomitant decrease in gastric secretion and, consequently, lower esophageal acid reflux. This allows the improvement of both GERD-related symptoms and esophageal diseases. One more point to be considered is that may or may not be related to HP are the factors that trigger the predisposition to EB. Among them, the genetic profile (for example IL-12p70) predisposing intense proinflammatory response, stimuli to bile salt reflux and risk factors (for example hiatal hernia), would be the driving force in the development of EB. Not least are the factors that can trigger HP infection, i.e., changes in gastric microbiota related to previous antibiotic use, diet and/or hygiene^8^ ^,^ ^24^ ^,^ ^25^ ^,^ ^28^food intake, and acid production, thereby decreasing weight and gastroesophageal reflux., METHODS: We evaluated the association of ghrelin with esophageal adenocarcinoma using a population from a previous nested case-control study. Among 128,992 enrolled in a multiphasic health checkup (MHC. Given many theories, hypotheses and possibilities, it is possible to agree with Malfertheiner et al.^22^ in part intriguing, and has become a matter of debate because of conflicting results. The cardia is an area where both H pylori and abnormal GERD exert their damaging potential, inducing inflammation and its consequences, such as intestinal metaplasia. While the role of intestinal metaplasia within columnar lined epithelium (Barrett’s oesophagus who have shown the need for further studies to prove the existence of any relationship between HP and GERD related esophageal diseases.

## CONCLUSION

There is an inverse relationship between HP and areas of distal esophageal columnar epithelialization, regardless of the presence or absence of intestinal metaplasia. Regarding age and gender, regardless of HP, it was noted that in women and older there is an increase in the number of cases with intestinal metaplasia in the distal esophagus.
